# Sex differences in renal angiotensin converting enzyme 2 (ACE2) activity are 17β-oestradiol-dependent and sex chromosome-independent

**DOI:** 10.1186/2042-6410-1-6

**Published:** 2010-11-05

**Authors:** Jun Liu, Hong Ji, Wei Zheng, Xie Wu, Janet J Zhu, Arthur P Arnold, Kathryn Sandberg

**Affiliations:** 1 Center for the Study of Sex Differences in Health, Aging and Disease, Georgetown University, Washington DC 20057, USA; 2 Department of Medicine, Georgetown University, Washington DC 20057, USA; 3 Department of Integrative Biology and Physiology and Laboratory of Neuroendocrinology, Brain Research Institute, University of California, Los Angeles, California 90095, USA

## Abstract

**Background:**

Angotensin converting enzyme 2 (ACE2) is a newly discovered monocarboxypeptidase that counteracts the vasoconstrictor effects of angiotensin II (Ang II) by converting Ang II to Ang-(1-7) in the kidney and other tissues.

**Methods:**

ACE2 activity from renal homogenates was investigated by using the fluorogenic peptide substrate Mca-YVADAPK(Dnp)-OH, where Mca is (7-methoxycoumarin-4-yl)-acetyl and Dnp is 2,4-dinitrophenyl.

**Results:**

We found that ACE2 activity expressed in relative fluorescence units (RFU) in the MF1 mouse is higher in the male (M) compared to the female (F) kidney [ACE2 (RFU/min/μg protein): M 18.1 ± 1.0 versus F 11.1 ± 0.39; *P *< 0.0001; *n *= 6]. Substrate concentration curves revealed that the higher ACE2 activity in the male was due to increased ACE2 enzyme velocity (V_max_) rather than increased substrate affinity (K_m_). We used the four core genotypes mouse model in which gonadal sex (ovaries versus testes) is separated from the sex chromosome complement enabling comparisons among XX and XY gonadal females and XX and XY gonadal males. Renal ACE2 activity was greater in the male than the female kidney, regardless of the sex chromosome complement [ACE2 (RFU/min/μg protein): intact-XX-F, 7.59 ± 0.37; intact-XY-F, 7.43 ± 0.53; intact-XX-M, 12.1 ± 0.62; intact-XY-M, 12.7 ± 1.5; *n *= 4-6/group; *P *< 0.0001, F versus M, by two-way ANOVA]. Enzyme activity was increased in gonadectomized (GDX) female mice regardless of the sex chromosome complement whereas no effect of gonadectomy was observed in the males [ACE2 (RFU/min/μg protein): GDX-XX-F, 12.4 ± 1.2; GDX-XY-F, 11.1 ± 0.76; GDX-XX-M, 13.2 ± 0.97; GDX-XY-M, 11.6 ± 0.81; *n *= 6/group]. 17β-oestradiol (E_2_) treatment of GDX mice resulted in ACE2 activity that was only 40% of the activity found in the GDX mice, regardless of their being male or female, and was independent of the sex chromosome complement [ACE2 (RFU/min/μg protein): GDX+E_2_-XX-F, 5.56 ± 1.0; GDX+E_2_-XY-F, 4.60 ± 0.52; GDX+E_2_-XX-M, 5.35 ± 0.70; GDX+E_2_-XY-M, 5.12 ± 0.47; *n *= 6/group].

**Conclusions:**

Our findings suggest sex differences in renal ACE2 activity in intact mice are due, at least in part, to the presence of E_2 _in the ovarian hormone milieu and not to the testicular milieu or to differences in sex chromosome dosage (2X versus 1X; 0Y versus 1Y). E_2 _regulation of renal ACE2 has particular implications for women across their life span since this hormone changes radically during puberty, pregnancy and menopause.

## Introduction

**Figure 1:**
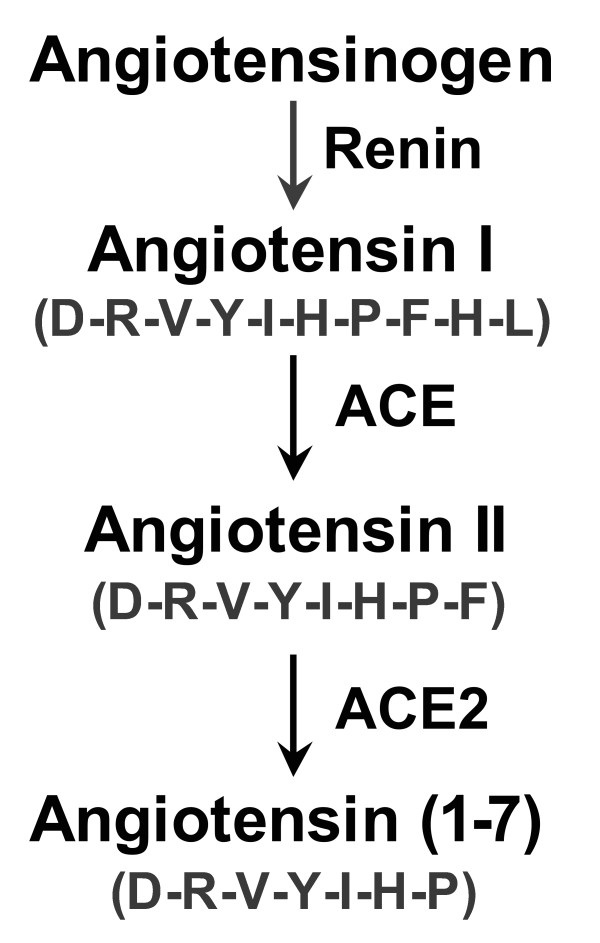
**Synthetic and catabolic pathways of Ang II**.

The discovery of angiotensin converting enzyme 2 (ACE2) is less than a decade old [1-3]. In contrast to angiotensin converting enzyme (ACE), which synthesizes the potent vasoconstrictor, angiotensin II (Ang II) through its dicarboxypeptidase activity acting on the decapeptide angiotensin I, ACE2 catabolizes Ang II through its monocarboxypeptidase activity (Figure 1). Studies suggest ACE2 counters the adverse effects of ACE action and that these two enzymes play a ying and yang role in the physiological regulation of Ang II metabolism and in disease pathologies such as hypertension, progressive renal disease and diabetes [1,4,5].

Much evidence indicates that Ang II metabolism is differentially regulated in males and females [6,7]. For example, plasma renin activity is 27% higher in men compared to women in a normotensive population [8] and plasma renin concentration (measured at pH 6.5) and serum ACE are significantly higher in male normotensive Lewis rats than in the females [9]. Studies also suggest Ang II metabolism is differentially regulated in the male and female kidney. In the normal rat kidney, no sex differences in renal ACE activity were observed. In contrast, renin concentration and neutral endopeptidase neprilysin were shown to be higher in the female than the male, whereas renal ACE2 was markedly lower in the female [9]. However, the cause of these sex differences in Ang II metabolism is not fully understood.

Sex differences in biology are due to the differences of being exposed *in utero *and through development to a male or female hormonal milieu. However, sex differences are also due to differences in sex chromosome dosage (2X versus 1X and 0Y versus 1Y). Fibroblast-based assays reveal expression heterogeneity among females; approximately 15%-25% of human X-linked genes escape X-inactivation fully or in part [10], although only a small subset of these genes show higher expression in females [11-13]. In mice, the percent of escape is less, but it still occurs and, thus, could contribute to sex differences in gene activity [13]. Another potential difference in the effects of the XX and XY sex chromosome complement arises from parental imprinting. While the X chromosomes in males are only imprinted maternally, the X chromosomes in females are imprinting maternally and paternally. Parental imprinting can exert major influences on gene function and have striking pathophysiological consequences [14,15].

It has been difficult to isolate the effects of the gonadal hormones from the effects of the sex chromosomes because the Y chromosome houses *Sry*, the testis-determining gene. However, in the four core genotypes (FCG) mouse model, separating effects of the gonads from the sex chromosomes is now possible [16]. In FCG mice, the Y^- ^chromosome lacks *Sry *[16] and, thus, XY^- ^mice develop ovaries. Inserting the *Sry *gene onto an autosome enabled the creation of the XY^-^*Sry *mouse with testes. Breeding XX female mice with XY^-^*Sry *male mice results in the FCG: XX and XY^- ^gonadal females and XX*Sry *and XY^-^*Sry *gonadal males.

Interestingly, ACE2 is located on the X chromosome [17], which raises the possibility that differences in sex chromosome dosage (2X versus 1X) could impact ACE2 activity due to escape from X-inactivation on the second X or differences in parental imprinting. As the kidney is one of the most abundant tissue sources of ACE2 [5], after optimizing an ACE2 assay for mouse tissues, we investigated the separate effects of the gonads and sex chromosomes on ACE2 in the kidney using the FCG.

## Methods

### Animals

The mice used in these studies included the random-bred MF1 wild type mouse, the FCG on the MF1 background and ACE2 null mice (ACE2^-/-^) on the C57BL/6 background. Studies were conducted in mice between 3-6 months old. MF1 breeders were purchased from Harlan Laboratories (MD, USA). The FCG breeders were received from Dr. Arthur P. Arnold's laboratory at UCLA (CA, USA). The ACE2^-/- ^mice originated from Dr. Susan Gurley and Thomas Coffman at Duke University (NC, USA) [18] but were received from Dr Eric. Lazartigues' laboratory at Louisiana State University (LA, USA). All mice were bred at Georgetown University as previously described [18,19]. Since mice from each litter were distributed across groups, prenatal and postnatal environment and litter effects were distributed across groups and genotypes. All mice were maintained on a phytoestrogen free diet (Harlan) and given tap water *ad libitum *under controlled conditions (12 h light/dark schedule at 24°C). Although the diet was phytoestrogen free, it should be noted that the tap water may have contained phytoestrogens although a recent study showed that more than 99% of the phytoestrogens are removed from waste water by the municipal treatment process [20]. All procedures were approved by the Georgetown University Animal Care and Use Committee.

### Gonadectomy and 17β-estradiol (E_2_) treatment

Gonadectomies were conducted between 53-60 days of age. Bilateral incisions were made under isoflurane anaesthesia (Baxter Healthcare Corp, IL, USA), the vascular supply was ligated and the gonads (either testes or ovaries) were removed. The muscle layer was sutured and the incisions closed with wound clips. In the sham-operated mice, the animals were subjected to surgery and the gonads were manipulated but left intact. At the time of gonadectomy, some GDX mice were treated with E_2 _(oestradiol) for 1 month as described previously [21] by inserting an E_2 _pellet (0.5 mg/90 days; Innovative Research of America, FL, USA) subcutaneously resulting in a dose of 5.6 μg/day.

### Tissue homogenates

At the time of sacrifice, the kidneys, heart and lung were removed and immediately homogenized in 5 vol/tissue wet weight of ice-cold lysis buffer (50 mM MES, 300 mM NaCl, 10 μM ZnCl_2_, 0.01% Bis, pH 6.5). Protease inhibitors were also included in lysis buffer: 0.50 μg/mL pepstatin A, 0.25 μg/mL leupeptin, 0.1 mg/mL bacitracin and 0.57 mmol PMSF (Sigma, MO, USA). After centrifuging the homogenate at 12,000 × g for 10 min at 4°C, the supernatant was removed, aliquoted in 20 μL fractions and frozen at -80°C. The protein content in sample lysates was measured using the Bio-Rad protein reagent (Bio-Rad Laboratories, CA, USA).

### ACE2 enzyme activity

Renal ACE2 activity was measured using the caspase-1 fluorogenic substrate 7-Mca-Tyr-Val-Ala-Asp-Ala-Pro-Lys(Dnp)-OH (R&D Systems, MN, USA), where Mca is (7-methoxycoumarin-4-yl)-acetyl and Dnp is 2,4-dinitrophenyl. Upon binding this substrate, ACE2 removes the COOH-terminal Dnp moiety via resonance energy transfer, unleashing the fluorescence inherent in Mca [22]. Heart and lung ACE2 activity were measured using the fluorogenic substrate Mca-Ala-Pro-Lys(Dnp)-OH (Anaspec, CA, USA).

Each well in a 96 well microtitre plate contained 70 μL of reaction buffer (1 M NaCl, 0.5 mM ZnCl_2_, 75 mM Tris, pH 7.5) in the presence of vehicle or inhibitors of ACE and/or ACE2, unless otherwise specified. Total peptidase enzyme activity was measured in the presence of vehicle (reaction buffer). Non-ACE activity was defined as peptidase activity measured in the presence of 10 μM captopril - an ACE inhibitor (Sigma Chemical Co, MO, USA). Nonspecific peptidase activity was defined as peptidase activity measured in the presence of 10 μM captopril and 10 μM MLN-4760 - an ACE2 inhibitor (gift from Millennium Pharmaceuticals, MA, USA). Specific ACE2 activity was defined as non-ACE activity minus nonspecific peptidase activity. Immediately after adding 10 μL of fluorogenic substrate, 20 μL of renal homogenate was added to each well in order to reach a final concentration of 30 μM substrate and 10 μg renal protein/100 μL/well, unless otherwise stated. Product formation was determined at 37°C by following the fluorescence as a function of time using a fluorescence plate reader (FLUOstar Omega, BMG LABTECH Inc, NC, USA) at an excitation wavelength of 320 nm and an emission wavelength of 410 nm.

In order to confirm assay specificity, we compared ACE2 activity in the kidneys of ACE2 wildtype (ACE2^+/+^) and knockout (ACE2^-/-^) mice [18]. Under these conditions, the amount of fluorescence obtained was less than 3% in the knockout female compared to the wild type mouse [relative fluorescence units (RFU)/min/μg protein: ACE2^+/+^, 13.9 ± 0.26 versus ACE2^-/-^, 0.420 ± 0.014; *P *< 0.001; *n *= 2/group]. Initial velocities were determined from the rate of fluorescence increase over the 50-100 min time course corresponding to the linear range of the assay. To further confirm the fluorescence assay for ACE2 activity, we contracted with the Wake Forrest University Hypertension Core Laboratory and measured the kinetics of hydrolysis of [^125^I]-Ang II (from 500 pM to 10 μM) to [^125^I]-Ang-(1-7) (Figure 1) in male and female MF1 renal homogenates using reverse-phase high-performance liquid chromatography to quantitate angiotensin peptides as previously described [23].

### ACE2 Western blot

ACE2 protein was determined by Western blot using an ACE2 goat polyclonal antibody (R&D Systems). Protein (80 μg) extracted from mouse whole kidney was heated (95°C, 10 min) in a Laemmli buffer, separated on Criterion precasting Tris-HCl gels (Bio-Rad Laboratories) and then blotted onto nitrocellulose. Nonspecific binding was blocked with nonfat dry milk (5%) before ACE2 protein was detected using a goat polyclonal antibody (R&D Systems, Inc) and a rabbit anti-goat horseradish peroxidase-coupled second antibody (Kirkegaard and Perry Laboratories, Inc, MD, USA). Visualization was carried out with chemiluminescence (LumiGLO peroxidase chemiluminescent substrate, Kirkegaard and Perry Laboratories, Inc) using X-ray film (CL-XPOSURE, Thermo Scientific, IL, USA). ACE2 protein was expressed in arbitrary units (AU) of immunoreactive ACE2 protein normalized to β-actin.

In order to confirm assay specificity, we compared ACE2 protein in the kidneys of ACE2^+/+ ^and ACE2^-/- ^mice [18]. Under these conditions, the amount of immunoreactive protein obtained was less than 0.9% in the knockout female compared to the wild type mouse [AU: ACE2^+/+^, 94.0 ± 4.0 versus ACE2^-/-^, 0.890 ± 0.36; *P *< 0.01; *n *= 2/group].

### ACE2 mRNA expression

ACE2 mRNA was determined in renal extracts by real-time polymerase chain reaction (PCR) using the ABI Prism 7700 Sequence Detection System (Applied Biosystems, CA, USA). Total RNA was extracted with TRIzol reagent (Invitrogen, CA, USA). First strand cDNA was made from total RNA using iScript cDNA synthesis kit (Bio-Rad Laboratories) with MMLV RNase H+ reverse transcriptase, oligo(dT) and random hexamers. The PCR reaction mixture included cDNA, TaqMan Universal PCR Master Mix (Applied Biosystems), forward and reverse primers (300 nM each) and 10 μM probe [*Forward primer: *5'-TCT GGG CAA ACT CTA TGC TGA CT-3'; *Reverse primer: *5'-GGC TGT CAA GAA GTT GTC CAT TG-3'; and *Probe: *6 FAM- CGG AAA GTT GTC TGC CAC CCC ACA-TAMRA]. PCR reactions without reverse transcription were included to control for contamination by genomic DNA. ACE2 mRNA was expressed in pg/μg total RNA normalized to 18 S ribosomal RNA.

In order to confirm assay specificity, we compared ACE2 mRNA in the kidneys of ACE2^+/+ ^and ACE2^-/- ^mice [18]. Under these conditions, the amount of mRNA obtained was less than 2% in the knockout female compared to the wild type mouse [pg/μg total RNA: ACE2^+/+^, 3.71 ± 0.46 versus ACE2^-/-^, 0.050 ± 0.005; *P *< 0.001; *n *= 2].

### Statistics

Data are expressed as means ± standard error of mean. Enzyme kinetics were analysed using Prism 4.0 (GraphPad Software Inc, CA, USA) to generate *V*_max _and *t*_1/2 _values from the Michaelis-Menten equation: *V *= *V*_max_[*S*]/([*S*] + *K*_m_), where *V *= velocity; *V*_max _= maximum velocity; [*S*] = substrate concentration; *K*_m _= Michaelis-Menten constant. *T*-tests were used to compare one independent variable. Two-way ANOVA and three-way ANOVA analysis were used to compare two and three independent variables, respectively (Sigma Stat Build 3.5.0.54, Systat Software Inc, IL, USA). Differences were considered statistically significant at *P *< 0.05.

## Results

### Dependence of mouse renal ACE2 activity on pH

**Figure 2:**
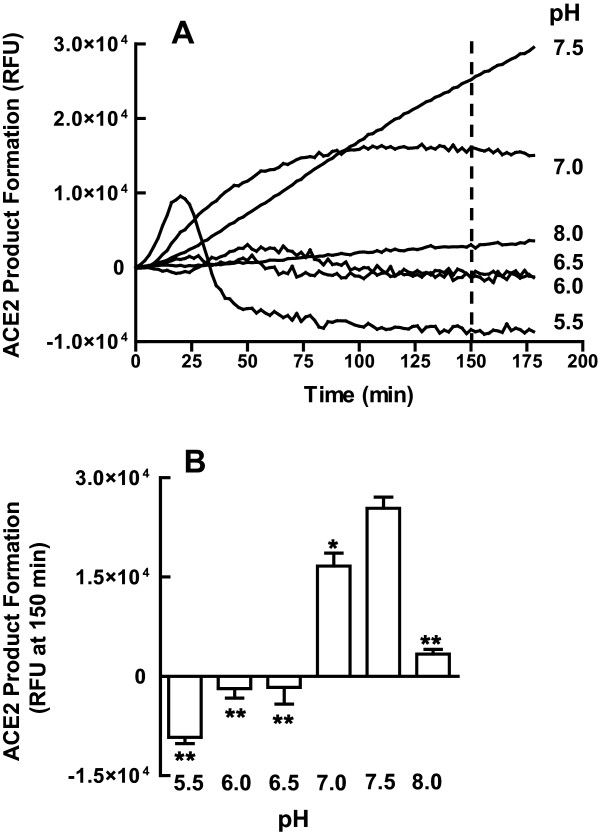
**Dependence of renal angiotensin converting enzyme 2 (ACE2) activity on pH in the MF1 mouse**. (A) Effect of pH on the time course of product formation in the female mouse. Data are representative of three experiments performed in triplicate. (B) Effect of pH on enzyme product formation at 150 min; *P < 0.05 and **P < 0.01 versus pH 7.5, *n *= 4/group.

ACE2 activity in female MF1 wild type mice renal homogenates was highly sensitive to pH and was optimal between pH 7.0 and 7.5 (Figure 2). The pH had a marked effect on the time course of enzyme product formation (RFU; Figure 2A). At pH 7.5, ACE2 product formation was linear with time up to 175 min, whereas at pH 7.0 product formation was linear only up to 50 min and at pH 5.5 the duration of the linear range was even shorter (25 min). This pH dependency of ACE2 activity is striking when product formation at 150 min is plotted as a function of pH (Figure 2B). Thus for all subsequent experiments, renal ACE2 activity was measured at pH 7.5. ACE2 enzyme activity in male renal homogenates was optimal between pH 7.0 and 7.5, similar to the data in the female (data not shown).

### Sexual dimorphism in dependence of mouse ACE2 activity on renal protein

**Figure 3:**
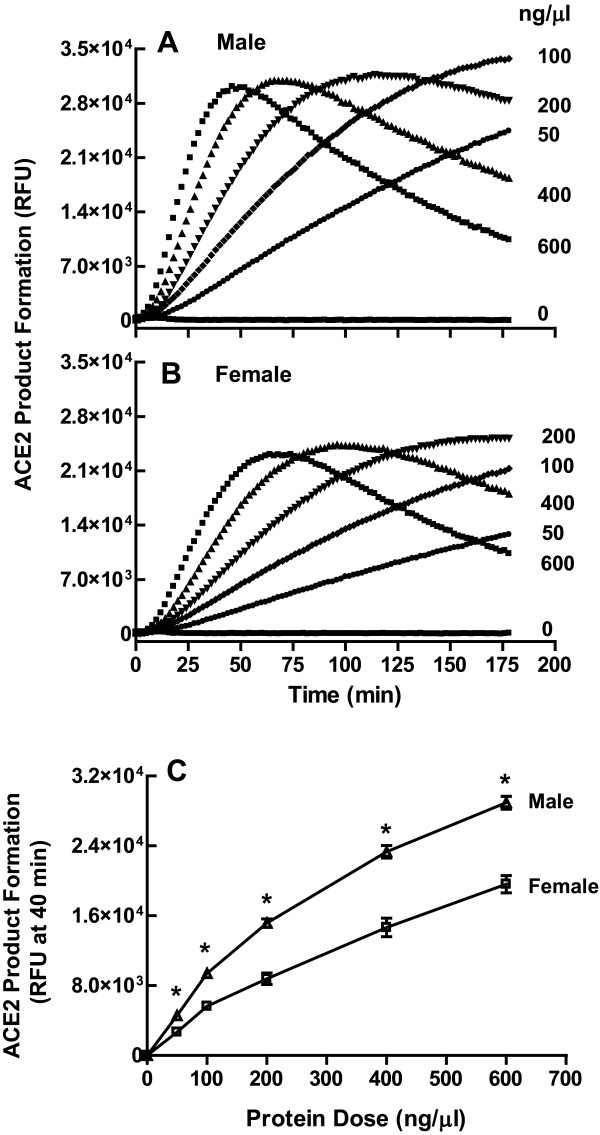
**Dependence of renal angiotensin converting enzyme 2 (ACE2) activity on protein concentration in the MF1 mouse**. Effect of protein concentration on the time course of product formation in (A) male and (B) female mice. Data are representative of three experiments performed in triplicate. (C) Effect of protein concentration on product formation at 40 min in male and female mice; *P < 0.01 versus female by *t*-test; two-way ANOVA revealed a significant effect of protein (*P *< 0.0001) and sex (P < 0.0001) on renal ACE2 activity; *n *= 4/group.

The relationship between renal ACE2 product formation and time was affected by the protein concentration in male (Figure 3A) and female (Figure 3B) MF1 wild type mice. There was a positive correlation between product formation and time up to 120 min for protein concentrations < 20 μg. However, this relationship became negative above this protein concentration. Thus, for all subsequent experiments, ACE2 activity in renal homogenates was measured using 10 μg of protein. Data from the 40 min reaction time point was collected and plotted in Figure 3C. Comparison of the protein dose response curves in males and females within this positive and linearly correlated range of the assay showed that ACE2 activity in the male kidney was 1.9-fold greater than the female (Figure 3C).

### Sexual dimorphism in kinetics of renal ACE2 activity

**Figure 4:**
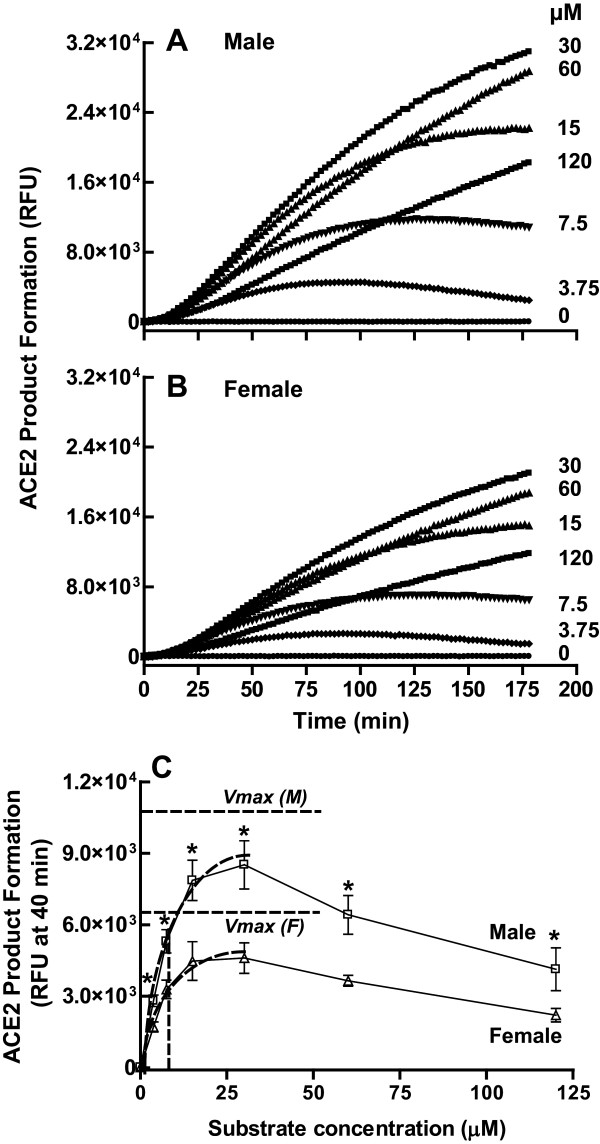
**Dependence of renal ACE2 activity on substrate concentration in the MF1 mouse**. Effect of substrate concentration on the time course of product formation in (A) male and (B) female mice. Data are representative of three experiments performed in triplicate. (C) Effect of substrate concentration on product formation at 40 min in male and female mice. Also shown is the determination of *K*_m _and *V*_max_; *P < 0.001 versus female by *t*-test; *n *= 4-5/group. Two-way ANOVA revealed a significant effect of substrate (*P *< 0.0001) and sex (*P *< 0.0001) on renal ACE2 activity.

The relationship between renal ACE2 activity and time was affected by the substrate concentration in male (Figure 4A) and female (Figure 4B) MF1 wild type mice. There was a positive correlation between enzyme activity and time up to 180 min for substrate concentrations < 30 μM. However, this relationship became negative above this substrate concentration in renal tissue from both sexes. Thus, for all subsequent experiments, renal ACE2 activity was measured using a substrate concentration of 30 μM. In order to determine whether the difference in renal ACE2 activity between males and females (Figure 3C) was due to differences in enzyme substrate affinity (*K*_m_) or to maximum enzyme velocity (*V*_max_), we calculated the *K*_m _and *V*_max _from ACE2 substrate dose response curves from 3.75 to 30 μM substrate concentration in male and female MF1 mice (Figure 4C). No sex differences were observed in the ACE2 *K*_m _[μM: male, 11.1 ± 0.93 versus female, 9.92 ± 0.56; not significant (ns); *n *= 4-5/group]. In contrast, the *V*_max _was 1.9-fold higher in the male kidney [RFU/min/μg: male, 34.5 ± 2.0 versus female, 18.4 ± 1.4; *P < 0.001; *n *= 4-5/group]. This sexual dimorphism in renal *V*_max _ACE2 activity, but not the *K*_m_, was confirmed by our findings using reverse-phase high-performance liquid chromatography to measure the enzyme kinetics of [^125^I]-Ang II metabolized to [^125^I]-Ang-(1-7) [*V*_max _(fmol/mg/min): male, 7.59 ± 0.63 versus female, 5.14 ± 0.55, *P *< 0.05; *K*_m _(μM): male, 3.43 ± 0.85 versus female, 3.36 ± 1.1, ns; *n *= 4/group].

### Comparison of ACE2 activity in male and female kidney, heart and lung tissues

**Figure 5:**
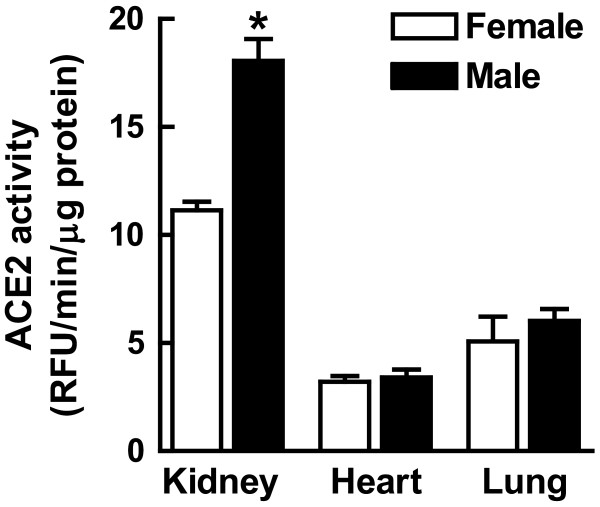
**Angiotensin converting enzyme 2 (ACE2) activity in the kidney, heart and lung of the MF1 mouse**. Comparison of ACE2 activity in male and female mice tissue homogenates using 10 μg kidney and 40 μg heart and lung protein and 30 μM substrate concentration for all three tissues; *P < 0.001 versus female by *t*-test; *n *= 5/group.

In order to determine whether the sex differences in renal ACE2 activity were specific to the kidney, we optimized an ACE2 assay in the heart and lung from MF1 wild type mice as these tissues are known to abundantly express ACE2 [5]. ACE2 activity was optimal at pH 7.5 and linear up to 80 μg protein for both myocardial and lung tissues (data not shown). There was a positive correlation between enzyme activity and time up to 180 min for substrate concentrations up to 30 μM. However, this relationship became negative above this point in both tissues. Therefore, we measured initial linear velocity (RFU/min μg protein) for ACE2 at 30 μM substrate for all three tissues (Figure 5).

Under these conditions, ACE2 activity was 1.6-fold higher in the male kidney [ACE2 (RFU/min/μg protein): male, 18.1 ± 1.0 versus female, 11.1 ± 0.39; *P *< 0.0001; *n *= 6]. In contrast, no significant differences in ACE2 activity between male and female mice were detected in the heart [ACE2 (RFU/min/μg protein): male, 3.41 ± 0.37 versus female, 3.22 ± 0.26; *n *= 5] and lung [ACE2 (RFU/min/μg protein): male, 6.03 ± 0.54 versus female, 5.07 ± 1.1; *n *= 5].

### Sexual dimorphism in renal ACE2 protein and mRNA expression

**Figure 6:**
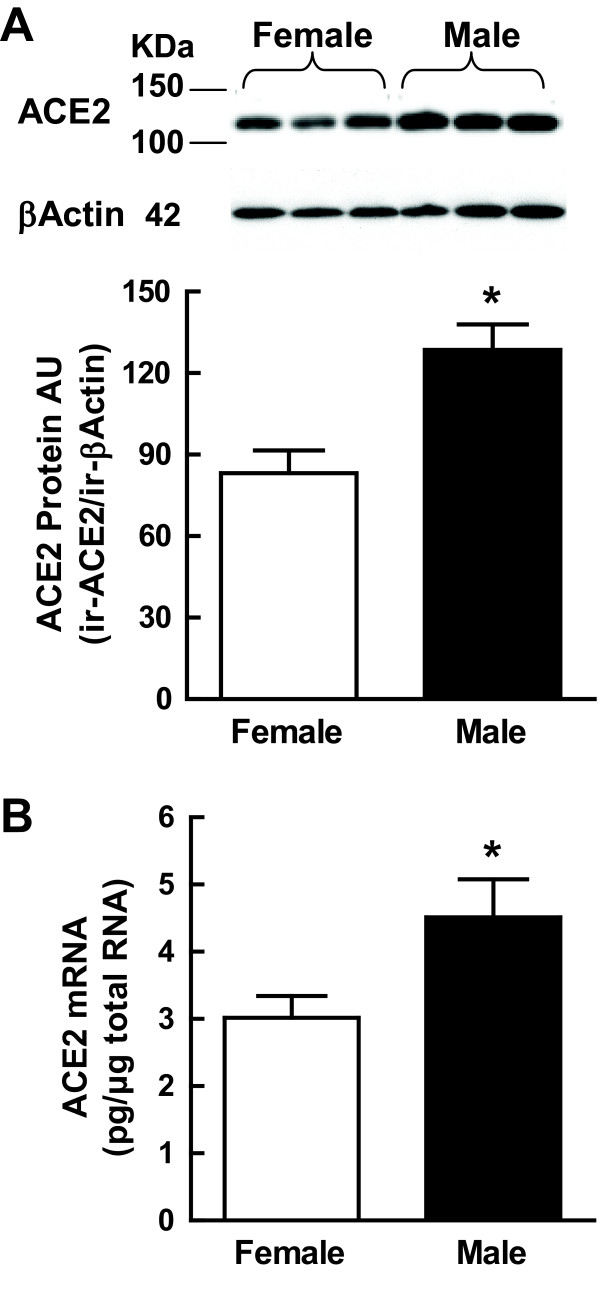
**Angiotensin converting enzyme 2 (ACE2) protein and messenger RNA (mRNA) expression in the MF1 mouse kidney**. (A) Quantitation of ACE2 protein expression from Western blots (upper) of male and female mouse renal homogenates; *P < 0.05 versus female; *n *= 7/group. (B) Quantitation of renal ACE2 mRNA expression determined by real-time polymerase chain reaction in male (*n *= 5) and female (*n *= 7) mouse kidney; *P < 0.05 versus female.

In order to determine whether the difference in renal ACE2 activity between males and females was due to differences in protein expression, we performed Western blot analysis of ACE2 in renal homogenates from male and female mice (Figure 6A). Immunoreactive renal ACE2 protein was expressed at a 1.5-fold higher level in males.

In order to determine whether the difference in renal ACE2 protein expression between males and females was due to differences in mRNA expression, we performed real-time PCR on renal ACE2 in male and female mice (Figure 6B). Renal ACE2 mRNA was expressed at a 1.5-fold higher level in male mice.

### Sex chromosome-independent ovarian and E_2_-mediated effects on renal ACE2 activity

**Figure 7:**
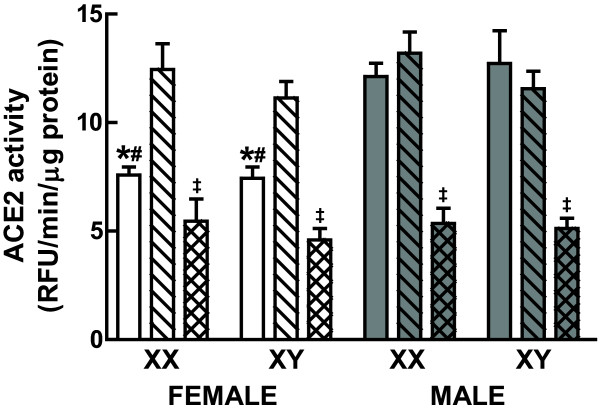
**Renal angiotensin converting enzyme 2 (ACE2) activity in the four core genotypes (FCG) in the intact and GDX state treated with and without oestradiol (E_2_)**. Shown are the mean ± standard error of mean for renal ACE2 activity in gonadally intact (open bars), GDX (striped bars) and E_2 _treated GDX (hatched bars) FCG mice; *n *= 6/group. ANOVA analyses revealed a significant effect of sex (*P *< 0.001, *intact-female versus intact-male), gonadectomy in the female (*P *< 0.001, #intact-female versus GDX-F) and E_2 _treatment in all GDX mice (*P *< 0.001, ^‡^GDX+E_2 _versus GDX) on renal ACE2 activity (see Results for details).

In order to determine the effect of gonadal sex (testes versus ovaries) independently of the sex chromosome complement (XX versus XY), we compared renal ACE2 activity in gonadally intact FCG at 4 months of age [ACE2 (RFU/min/μg protein): intact-XX-F, 7.59 ± 0.37; intact-XY-F, 7.43 ± 0.53; intact-XX-M, 12.1 ± 0.62; intact-XY-M, 12.7 ± 1.5; *n *= 4-6/group; Figure 7]. Analysis by two-way ANOVA (factors of sex, male versus female and sex chromosome complement, XX versus XY) showed that regardless of being XX or XY, intact males had approximately 1.6-fold higher renal ACE2 activity than intact females (*P *< 0.0001) and there was no gonad-independent effect of the sex chromosome complement on enzyme activity (Figure 7).

In order to determine the effect of gonadectomy independently of the sex chromosome complement, we measured renal ACE2 activity 1 month after gonadectomy in the FCG [ACE2 (RFU/min/μg protein): GDX-XX-F, 12.4 ± 1.2; GDX-XY-F, 11.1 ± 0.76; GDX-XX-M, 13.2 ± 0.97; GDX-XY-M, 11.6 ± 0.81; *n *= 6/group; Figure 7]. Analysis by three-way ANOVA (factors of sex, male versus female; sex chromosome complement, XX versus XY and, gonad state, GDX versus intact) showed that there was no effect of gonadectomy or the sex chromosomes on ACE2 activity in the males whereas the GDX female mice had approximately 1.6-fold higher renal ACE2 activity than the intact females (*P *< 0.001) regardless of being XX or XY.

In order to determine the effect of 17β-estradiol (E_2_) treatment in GDX mice independently of the sex chromosome complement, we measured the renal ACE2 activity after treating GDX mice with E_2 _(5.6 μg/day) for 1 month [ACE2 (RFU/min/μg protein): GDX+E_2_-XX-F, 5.56 ± 1.0; GDX+E_2_-XY-F, 4.60 ± 0.52; GDX+E_2_-XX-M, 5.35 ± 0.70; GDX+E_2_-XY-M, 5.12 ± 0.47; *n *= 6/group; Figure 7]. Analysis by three-way ANOVA (factors of sex, male versus female; sex chromosome complement, XX versus XY and E_2 _treatment, GDX versus GDX+E_2_) showed that E_2 _reduced renal ACE2 activity by 56%-59% compared to the GDX mice and that the effect of E_2 _(*P *< 0.001) was independent of sex and the sex chromosomal complement.

## Discussion

In this study, we initially optimized a fluorescent ACE2 assay for the mouse kidney. ACE2 activity was optimal at a pH of 7.5. Major reductions in enzyme activity occurred at 0.5 units above or below this pH. Using this same substrate, recombinant soluble human ACE2 has previously been shown to be strongly pH dependent, though the optimal pH for human ACE2 was 6.5 [24]. Assaying ACE2 in mouse renal homogenates at a pH of 6.5 resulted in > 95% inhibition of the maximal ACE2 activity. Thus, the pH optimum for ACE2 activity is dependent upon the species (human versus mouse) or on the source of the enzyme (recombinant versus renal homogenates). This discrepancy in pH dependence may be an important consideration when comparing wild type ACE2 activity in mice to ACE2 activity in mice overexpressing the human form of the enzyme [25]. This enzyme's strong pH sensitivity may reflect the importance of Arg^273 ^and His^345^, which are two basic residues in ACE2 that are highly conserved across species including *Drosophiila*, rabbit, mouse, rat and human [2]. Arg^273 ^plays a key role in substrate affinity and His^345^, by acting as a hydrogen bond donator/acceptor in the peptide intermediate, plays a critical role in the enzymatic reaction [26].

Renal homogenates above 20 μg protein resulted in a loss of assay linearity with respect to time, suggesting that some tissue factor(s) achieves sufficient concentration to inhibit the assay above this amount. This factor may be similar to the small, hydrophilic and cationic endogenous inhibitor of ACE2 that was recently found in human plasma [27].

The ACE2 enzyme was subject to substrate inhibition at substrate concentrations above 30 μM. This phenomenon happens for 20% of all known enzymes and is usually due to the binding of two substrate molecules to the enzyme [28]. At low substrate concentrations, only one substrate molecule binds and normal enzyme kinetics occur. In contrast, when a second substrate binds due to excess substrate, enzyme catalysis is often inhibited. Thus, some studies of ACE2 activity in mouse kidney in which 100 μg of renal homogenates [29] or 100 μM substrate [30] were used may be confounded by the presence of tissue inhibitors or substrate inhibition.

Under these assay conditions, we found that ACE2 activity is sexually dimorphic in the kidney. Male mice have higher levels of renal ACE2 activity. This difference in activity is due to a higher *V*_max _in the male compared to the female kidney and not to sex differences in the affinity of the substrate for the enzyme. These findings support a previous study in normotensive Lewis rats, which showed that ACE2 activity in the male renal cortex was higher when compared to the female rat [9]. It will be interesting to determine if this sexual dimorphism in renal ACE2 activity is tightly conserved across mammalian species including humans or whether it is specific to rodents.

Although ACE2 activity is higher in the male kidney under normal conditions, it does not necessarily mean this would be the case under pathological conditions. Studies have shown that renal ACE2 is up- [31] and down-regulated [32] in disease pathologies. Given our finding of sex differences in basal renal ACE2 activity, it is imperative to determine if there is also sexual dimorphism in renal ACE2 regulation under pathological conditions.

Apparently, this sex difference in renal ACE2 activity is not found in all tissues expressing the enzyme since no significant differences in myocardial or lung ACE2 activity were observed in these same male and female mice under our experimental conditions. The lack of sexual dimorphism in mouse heart and lung ACE2 activity is similar to the findings of studies of cardiac ACE2 in the Lewis rat [9] and to lung ACE2 in the Sprague Dawley rat [33], since no differences in ACE2 activity between males and females were observed in these tissues. Our findings of tissue-specific sexual dimorphism in renal ACE2 activity also support a report that showed that ovarian hormones down-regulated ACE2 mRNA expression in the mouse kidney but not in the lung [34].

The higher ACE2 *V*_max _in male kidneys compared to females is likely due to higher levels of renal ACE2 protein expression since male kidneys expressed more renal ACE2 protein than the female kidneys. The similarity in the sex difference ratio (male:female) between protein (1.5-fold) and mRNA (1.5-fold) suggests sex differences in ACE2 protein expression are a direct result of sex differences in the rate of ACE2 mRNA transcription. Thus, these findings also suggest that renal ACE2 is differentially regulated at the transcriptional level in males and females. Interestingly, the sex difference ratio for enzyme activity (1.9-fold) was higher than the sex difference ratio for protein and mRNA expression. This finding raises the possibility that the enzyme is differentially modified after protein synthesis in males and females in such a way as to alter the enzyme activity since the sex difference ratio for enzyme activity is amplified by 1.3-fold compared to the sex difference ratio in protein or mRNA. The resolved crystal structure of ACE2 [35] suggests six of the seven potential sites [3] are glycosylated, although there are no published studies to date that explain how glycosylation alters enzyme activity. Recently, ACE2 has been shown to bind calmodulin through a calmodulin binding domain within the cytoplasmic tail. Upon binding calmodulin, ACE2 ectodomain shedding occurs which leads to as increased release of the catalytically active soluble form of the enzyme [36]. However, there are no reports to date on how calmodulin binding affects ACE2 activity in the kidney.

The major finding of our study was that sex differences in renal ACE2 activity are caused by the female-specific effects of the ovary and in particular, the presence of E_2 _and the fact that this effect is independent of the sex chromosome complement or gonadal sex of the animal. Previously, the effects of the gonads were not separable from the effects of the sex chromosomes. That is, regulation of ACE2 activity by gonadectomy could not rule out the possibility that the effect of gonadectomy was really an effect of differences in X or Y dosage that were unmasked in the absence of the gonads. The FCG model, however, enables dissection of potential gonadal effects from potential sex chromosome effects. Thus, we were able to unequivocally demonstrate that ACE2 activity is higher in the male kidney compared to the female because of the presence of ovaries and the ovarian hormone E_2 _and not because of the differences in sex chromosome dosage (2X versus 1X or 0Y versus 1Y). As ACE2 expression was not higher in mice with two X chromosomes relative to mice with one X chromosome, the findings suggest that ACE2 does not escape X-inactivation, at least not in the mouse tissues examined [13]. It remains possible, however, that ACE2 is regulated by X chromosome dosage in humans since there are a greater number of genes that escape X-inactivation in the human X chromosome [10]. Furthermore, the lack of a difference between GDX gonadal males and females, and their equal response to oestradiol as adults, suggest that there are no major organizational effects of gonadal hormones on renal ACE2 activity. Apparently, sex differences in the *in utero *hormonal milieu or in the hormonal milieu during prepubertal development do not permanently alter renal ACE2 activity. Moreover, the fact that E_2 _treatment lowered ACE2 activity in GDX male mice to the same extent as GDX female mice indicates the this attenuating effect of E_2 _on renal ACE2 activity is independent of any organizational effects resulting from sex differences in the *in utero *hormonal milieu.

Previously, we found that ovariectomy attenuated renal ACE2 activity in a rat model of progressive renal disease and that this effect was prevented by E_2 _treatment [37]. The finding that renal ACE2 is differentially regulated by E_2 _under normal and disease conditions suggests the enzyme plays different physiological roles in the normal and disease state. It would be interesting in future studies to determine if compensatory actions of ACE2 that have been described in disease states, such as diabetes [4], is sexually dimorphic. Do males up-regulate renal ACE2 to a greater extent than females in renal disease states? Is this a necessary compensation for the greater renal injury experienced by the male kidney to the same insult? It is known that the male kidney is more susceptible to renal disease pathology than the female in humans [38] and in animal models [39]. Alternatively, could sexual dimorphism in the ability to up-regulate ACE2 contribute to the sex differences in renal injury [40]?

Ovarian and, in particular E_2 _regulation of renal ACE2, raises the possibility that the enzyme is differentially regulated in women across their life span since the ovarian hormone milieu radically changes from birth to menarche, during pregnancy and through the menopause transition. In fact, renal ACE2 activity has been shown to be upregulated during pregnancy in Sprague Dawley rats [41]. Thus, dysregulation of the ovarian hormone milieu may increase susceptibility for diseases involving the renin angiotensin system and may have particular repercussions for postmenopausal women and women with premature ovarian failure. However, it is important to note that, although we did not find that removing the testes impacted renal ACE2 activity, this study can not rule out the possibility that ACE2 in male kidneys is regulated by other factors associated with development and ageing.

This study was conducted in a mouse model that does not carry any known mutations that increase susceptibility to disease. Thus, it remains possible that the mechanisms underlying the sex differences in renal ACE2 activity disappear or are augmented by pathophysiology. In fact, we previously reported that, in the renal wrap model of hypertension-associated renal disease, ovariectomy decreased renal ACE2 activity and protein expression while E_2 _replacement prevented these effects in the Sprague Dawley rat [37]. Furthermore, E_2 _treatment of ovariectomized Sprague Dawley rats was shown to increase the levels of cardiac ACE2 protein expression under conditions of deocycorticosterone acetate-induced hypertension and cardiac remodelling [42]. In the pathophysiological state, where a role for ACE2 has been implicated, sex differences in the degree of injury-induced down-regulation of ACE2 or in the ability to up-regulate ACE2 as a defence mechanism could be key factors that have major implications for diabetes [4] and other disease pathologies such as hypertension [1].

## Conclusion

After optimizing an ACE2 assay in mouse tissue, this study found that the sex difference in basal ACE2 activity is selective for the kidney; males have higher renal ACE2 activity than females whereas, under normal conditions, no sex differences in ACE2 activity were detected in the heart and lung. The higher renal ACE2 activity in males is probably due to sex differences in both post-transcriptional and post-translational mechanisms of gene regulation. Most importantly, our study demonstrates unequivocally that ACE2 is lower in the female kidney because of the effects of the ovary and is due, at least in part, to the presence of E_2 _and not because of testicular effects or the sex chromosome complement. These findings suggest new therapeutics that target ACE2 need take into account the ovarian hormone milieu across the female lifespan.

## Abbreviations

ACE: angiotensin converting enzyme; AngII: angiotensin II; AU: arbitrary unit; E_2 _= 17β-estradiol; FCG: four core genotype; PCR: polymerase chain reaction; RFU: relative fluorescence unit.

## Competing interests

The authors declare that they have no competing interests.

## Authors' contributions

JL participated in the experiments design, carried out the enzymatic activity studies, performed the statistical analysis and drafted the manuscript. HJ participated in the experiments design and performed real time PCR. WZ and XW carried out the animal experiment, including surgery. JJZ carried out the immuno-blot. APA participated in the experiments design and data analysis. KS conceived the study, participated in its design and coordination and wrote the manuscript. All authors read and approved the final manuscript.
